# Unravelling the effect of data augmentation transformations in polyp segmentation

**DOI:** 10.1007/s11548-020-02262-4

**Published:** 2020-09-28

**Authors:** Luisa F. Sánchez-Peralta, Artzai Picón, Francisco M. Sánchez-Margallo, J. Blas Pagador

**Affiliations:** 1 Jesús Usón Minimally Invasive Surgery Centre, Road N-521, km 41.8, 10071 Cáceres, Spain; 2 Tecnalia Research and Innovation, Zamudio, Spain

**Keywords:** Polyp segmentation, Deep learning, Data augmentation, Transformations, Semantic segmentation

## Abstract

**Purpose:**

Data augmentation is a common technique to overcome the lack of large annotated databases, a usual situation when applying deep learning to medical imaging problems. Nevertheless, there is no consensus on which transformations to apply for a particular field. This work aims at identifying the effect of different transformations on polyp segmentation using deep learning.

**Methods:**

A set of transformations and ranges have been selected, considering image-based (width and height shift, rotation, shear, zooming, horizontal and vertical flip and elastic deformation), pixel-based (changes in brightness and contrast) and application-based (specular lights and blurry frames) transformations. A model has been trained under the same conditions without data augmentation transformations (baseline) and for each of the transformation and ranges, using CVC-EndoSceneStill and Kvasir-SEG, independently. Statistical analysis is performed to compare the baseline performance against results of each range of each transformation on the same test set for each dataset.

**Results:**

This basic method identifies the most adequate transformations for each dataset. For CVC-EndoSceneStill, changes in brightness and contrast significantly improve the model performance. On the contrary, Kvasir-SEG benefits to a greater extent from the image-based transformations, especially rotation and shear. Augmentation with synthetic specular lights also improves the performance.

**Conclusion:**

Despite being infrequently used, pixel-based transformations show a great potential to improve polyp segmentation in CVC-EndoSceneStill. On the other hand, image-based transformations are more suitable for Kvasir-SEG. Problem-based transformations behave similarly in both datasets. Polyp area, brightness and contrast of the dataset have an influence on these differences.

**Electronic supplementary material:**

The online version of this article (10.1007/s11548-020-02262-4) contains supplementary material, which is available to authorized users.

## Introduction

Deep learning techniques have been widely used for the last years as they have proved their ability to extract features for different computer vision tasks such as object detection, classification or segmentation [1]. Undoubtedly, these techniques have also been used for medical imaging with great success [2, 3]. Even though, one limitation that must be faced in this field is the lack of large datasets with relevant annotations and/or labelling [4, 5]. One of the most widely used strategies for addressing this problem is data augmentation [6].

Data augmentation for images consists of increasing the amount and diversity of training cases based on the available images in the database through the application of image transformations such as translation or flipping of the original image [7]. Different computational libraries have been created to perform these transformation functions [8, 9]. However, the selection of the most suitable strategy remains a trial-and-error process that depends on the experience, imagination and time of the researcher [10]. There are several studies analysing the effect of data augmentation for image classification tasks [11–14], but this field is not fully explored for semantic segmentation yet [15].

Computer-assisted diagnosis (CAD) systems for early detection of colorectal cancer have also benefited from the application of deep learning techniques [16–18]. Publicly available datasets range from hundreds of images with a manually segmented binary mask, such as CVC-EndoSceneStill [19] or Kvasir-SEG [20], to thousands of video frames with an approximated elliptical binary mask, such as CVC-VideoClinicDB [21, 22]. For polyp segmentation, it is easy to find several works in which data augmentation has been used. Nevertheless, there is a wide variety of transformations selected as well as their ranges (for example, rotating between − 45° and 45° instead of between − 90° and 90°). Table 1 gathers the applied transformations and their ranges, when available, for recent works on polyp segmentation using deep learning. Although there are authors who do use data augmentation, they do not describe the transformations applied [23]. Besides, it is also important to point out that more intense data augmentation does not necessarily yield to increased performance [24]. The particularities of the medical image type must also be taken into consideration for selecting data augmentation transformations, as the image might have particularities that affect image processing methods. For polyp segmentation, specular lights negatively affect detection methods as they prominently appear, hiding colour and textural information [25].

**Table 1:** Transformations used for data augmentation in polyp segmentation

Work	Year	Rotation	Width shift	Height shift	Shear	Zoom	Flip	Warp	Gaussian noise	Contrast	Brightness	Patch selection
Jha [20]	2020		–	–	–	✓	✓	–	–	–	✓	–
Guo [26]	2019	✓	–	–	–	✓	✓	–	–	–	✓	–
Kang [27]	2019	(− 45°, 45°)	–	–	(− 16°, 16°)	(0.5, 1.5)	✓	–	–	(0.5, 1.5)	(0.8, 1.5)	✓
Akbari [28]	2018	10° interval, between 0°–290°	–	–	–	–	✓	–	–	–	–	15 patches/image
Brandao [29]	2018	–	–	–	–	–	✓	–	–	–	–	224 × 224 patches
Wichakam [30]	2018	up to 180°	(0, 20%)	(0, 20%)	up to 20%	(–0.8, 1.2)	✓	–	–	–	–	–
Wickstrom [31]	2018	(–90°, 90°)	–	–	(0, 0.4)	(0.8, 1.2)	–	–	–	–	–	224 × 224 patches
Bardhi [32]	2017	✓	✓	✓		–	✓	–	✓	–	–	✓
Li [33]	2017	✓	✓	✓	–	–	–	–	✓	✓	–	–
Vázquez [19]	2017	(0°, 180°)	–	–	(0, 0.4)	(0.9, 1.1)	–	(0, 10)	–	–	–	–

We hypothesize that the application of different transformations as well as different ranges for the same transformation might lead to differences in performance. Thus, the objective of this work is to elucidate the effect of different image transformations and their ranges used for data augmentation for polyp segmentation. Therefore, this work does not pursue to obtain the best segmentation results but to analyse how the different transformations and their ranges used in data augmentation might influence the results of polyp segmentation in endoscopic images using deep learning.

## Methods

### Transformations

**Fig. 1:**
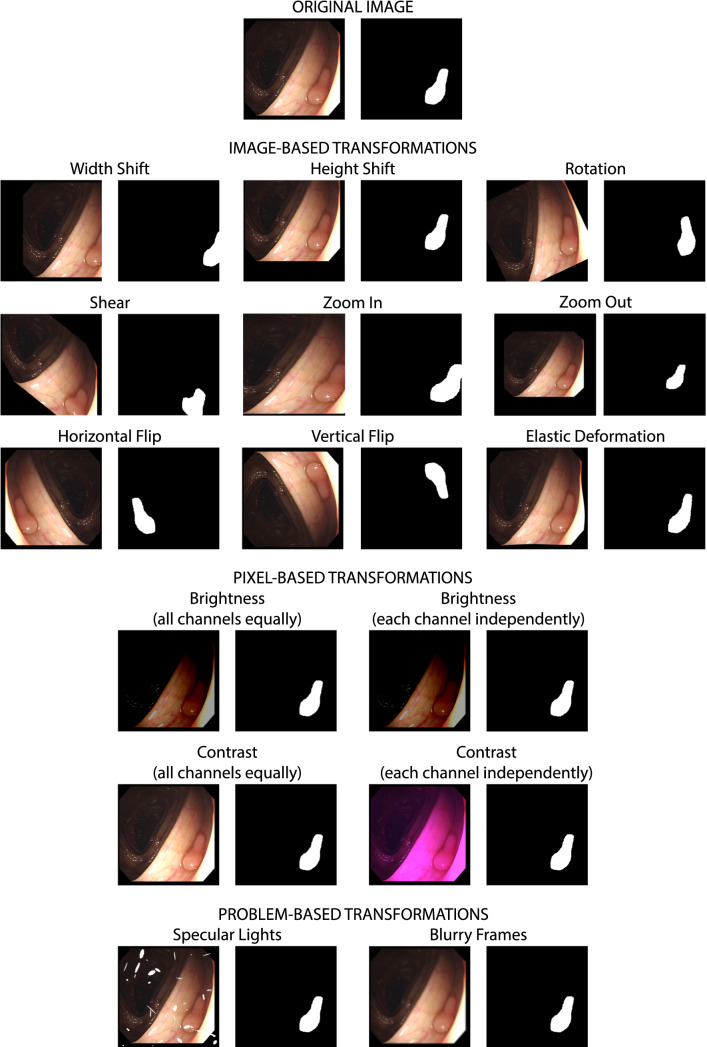
Original and transformed images

Different transformations have been considered in this study, which can be classified into three categories. For each transformation, a suitable range of values has been established (Table 2). Figure 1 shows an example of the result of applying each transformation to an image. In the case of image-based transformations, image and mask are transformed in the same way.

**Table 2:** Transformations and ranges analysed in this study

Transformation	Parameter definition	Ranges	Total cases
Image-based transformations			
Width shift	% of the image displaced to the right or to the left	0–90%, with 10% intervals	9 cases
Height shift	% of the image displaced up or down	0–90%, with 10% intervals	9 cases
Rotation	± Degrees that the image is rotated	0–180°, with up to 45° intervals	8 cases
Shear	± Shear angle in counter-clockwise direction	0–180°, with up to 45° intervals	8 cases
Zoom out	Factor by which the image size is multiplied	1 − *x*, *x* ∈ [0.1, 0.9], with 0.1 intervals	9 cases
Zoom in	Factor by which the image size is multiplied	1 + *x*, *x* ∈ [0.1, 1.0], with 0.1 intervals	10 cases
Flip	Vertically and horizontally flip the image	True	2 cases
Elastic deformation	Parameters as indicated in [32]	*α* values: 250, 500, 1000, 2000, 3000, 4000, 5000, 6000 *σ* value: fixed at 40	8 cases
Pixel-based transformations			
Brightness	± value to be added to the actual pixel value for all RGB channels equally	[25, 175], with 25 intervals	7 cases
Brightness	Value to be added to the actual pixel value for each RGB channel independently	[25, 175], with 25 intervals	7 cases
Contrast	Value to multiply the actual pixel value for all RGB channels equally	[1 − *x*, 1 + x*x*, *x* ∈ [0.2, 1.0], in intervals of 0.2	5 cases
Contrast	Value to multiply the actual pixel value for each RGB channel independently	[1 − *x*, 1 + *x*], *x* ∈ [0.2, 0.8], in intervals of 0.2	4 cases
Application-based transformations			
Specular lights	Overexposed light ellipses simulating the effect of bright points	True	1 case
Blurry images	Window size of a mean filter	[1, 15], only even integers	7 cases

To model the specular lights, the CVC-EndoSceneStill database [19] has been used, as it provides a manually segmented class for specular lights in endoscopic images. Specular lights are modelled as ellipses of variable size and orientation. Size of major and minor axes are obtained from the specular lights in CVC-EndoSceneStill database, corresponding to a mean major axis of 7.77 ± 10.36 pixels (range 0–259.81) and a mean minor axis of 3.82 ± 4.29 pixels (range 0–137.39). The number of specular lights per image is modelled as a positive left-skewed distribution, with mean 18.20 and standard deviation 16.97, according to the distribution of CVC-EndoSceneStill. In the image, pixel values are set to 255 in all channels to create the ellipses according to the previously described distributions, with random locations on the image.

### Datasets, architecture and training process

Two publicly available datasets have been used in this work. CVC-EndoSceneStill [19] contains 912 images obtained from 44 video sequences collected from 36 patients. It explicitly indicates the images belonging to the training, validations and test sets. In this work, this division has been used. This way, all experiments use the same images, which allows for a fair comparison of performance. The training, validation and test sets comprise 547, 183 and 182 images, respectively. The second dataset is Kvasir-SEG [20]. It provides 1000 polyp images. The dataset has been divided into training, validation and test sets (800, 200 and 200 images, respectively), as this division is not provided by the dataset’s owners. Both datasets provide binary masks for each polyp image, where pixels corresponding to the class are labelled with 1, and 0 otherwise. Each dataset is used on its own to replicate the same experiments for further comparison of results. Table 3 shows some characteristics of the images included in the test sets of the datasets. Kvasir-SEG presents bigger polyps than CVC-EndoSceneStill, with images that are brighter and with more contrast and where the void area is smaller.

**Table 3:** Details for the datasets used in this study

	CVC-EndoSceneStill	Kvasir-SEG
Void area (%)	23.73 ± 5.57 (27.83–14.62)	15.23 ± 4.82 (28.44–6.16)
Polyp area relative to the valid area (%)	12.50 ± 11.49 (66.15–0.75)	17.36 ± 15.65 (83.66–0.61)
Mean value of brightness channel in HSV [34]	0.560 ± 0.006 (1.000–0.000)	0.622 ± 0.003 (1.000–0.000)
Histogram flatness measure [35]	0.858 ± 0.121 (0.959–0.000)	0.419 ± 0.443 (0.962–0.000)
Histogram spread [35]	0.252 ± 0.088 (0.520–0.076)	0.218 ± 0.070 (0.432–0.075)

Results are reported as mean ± standard deviation. Minimum and maximum values are indicated between brackets. The void area refers to the black area in the images, while the remaining area is considered as valid area

**Fig. 2:**
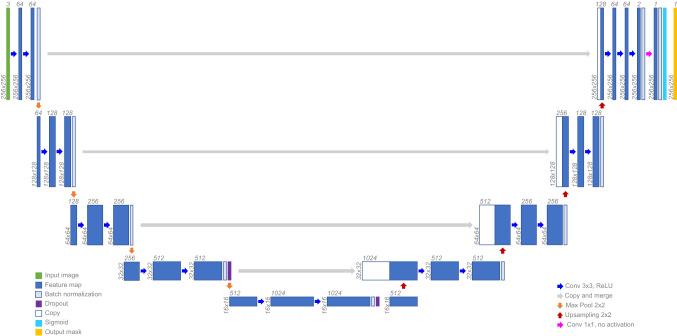
Network architecture. Figure based on [36]

Our network architecture (Fig. 2) is based on a U-Net architecture [36]. The down-sampling path transforms the input image of size 256 × 256 × 3 to a feature map of 16 × 16 × 1024 by applying five convolutional blocks. These blocks consist of two 3 × 3 convolutional layers, each one with a rectified linear unit, and a 2 × 2 max pool layer, except for the last block. The up-sampling path includes four blocks that produce a 256 × 256 × 1 probability map. Each block starts with a 2 × 2 up-sampling layer followed by a 3 × 3 convolutional layer, to whose result the corresponding feature map from the down-sampling path is concatenated. Zero padding preserves sizes along convolutional layers. We included batch normalization both in down- and up-sampling paths.

The network has been implemented using Keras [37] and Tensorflow [38] as backend. Experiments were run on a NVIDIA GTX 1080 GPU with 8 GB memory. The network has been pretrained using CVC-VideoClinicDB [21, 22], whose polyp masks are not precise but approximated to elliptical shapes. The datasets in “Transformations” section are then used to finetune this pretrained model with fixed parameters for all experiments:

- Adam optimizer, with default parameters in Keras: amsgrad = false; beta_1 = 0.9 and beta_2 = 0.999
- Learning rate: starting at 10^–4^, decreasing to half each epoch and recovering to 10^–4^ each 5 epochs
- 15 epochs
- Batch size: 4
- Image input size: 256 × 256 × 3
- Dropout: 0.5

Each experiment has been repeated ten times to minimize the effect of randomly applying transformations. Results are shown in terms of mean ± standard deviation of the mean. A baseline level has been established by finetuning the model without applying any data augmentation.

Since semantic segmentation is performed through a pixel-wise classification, we face an unbalanced dataset where the negative class (no polyp) is more present than the positive one (polyp) in each image. Therefore, the selected loss function combines the binary cross-entropy and the Jaccard index as in [39]:

$$ {\text{Loss}} = - \frac{1}{n}\sum\limits_{i,j} {\left( {y_{i,j} \log \hat{y}_{i,j} + \left( {1 + \hat{y}_{i,j} } \right)} \right)\log \left( {1 - \hat{y}_{i,j} } \right)} - \log J, $$

where the first term corresponds to the binary cross-entropy, being *y*_*i*,*j*_ the ground truth class for pixel (*i*, *j*) and $\hat{y}_{i,j}$ the predicted class; and *J* is the Jaccard index or Intersection over Union (IoU) defined as a similarity measure between sets *A* and *B* as:

$$ J = IoU\left( {A,B} \right) = \frac{{\left| {A \cap B} \right|}}{{\left| {A \cup B} \right|}} = \frac{{\left| {A \cap B} \right|}}{{\left| A \right| + \left| B \right| - \left| {A \cap B} \right|}}, $$

where $\left| X \right| = \sum\nolimits_{i} {x_{i} }$ being *x*_*i*_ is the i-th element of set *X*; ∩ is the intersection of sets and ∪ is the union of sets.

### Statistical analysis

Results of the ten repetitions have been statistically analysed to identify differences between distributions, using R (version 3.6.1) and RStudio (version 1.2.5033). Permutation test [40] is selected as no assumption on the distributions is required. In the permutation test, firstly the “observed mean” is calculated as the difference between means for the baseline and the group under study. Data are then shuffled and randomly assigned to each group and the corresponding “calculated mean” is obtained as the difference between means of the two groups. After 10000 repetitions, the *p* value is determined as the percentage of calculated means which are greater than the observed mean. Significance is evaluated at *p* value < 0.05, *p* value < 0.01 and *p* value < 0.001. This analysis is performed for each dataset independently.

## Results

For both datasets, Table 4 shows the results for the baseline and all transformations and ranges, together with the results of the permutation test to establish statistically significant differences between baseline and transformations.

**Table 4:** Mean and standard deviation of the mean for transformations and ranges analysed in both datasets

Transformation	Range	IoU on test set CVC-EndoSceneStill	IoU on test set Kvasir-SEG
None	N/A	59.10 ± 9.35	66.45 ± 8.08
Image-based transformations			
Width Shift	± 10%	60.78 ± 8.99	67.09 ± 7.96
	± 20%	59.45 ± 9.80	**67.34 ± 8.06**
	± 30%	59.31 ± 9.08	66.28 ± 8.22
	± 40%	62.70 ± 8.57	65.94 ± 8.22
	± 50%	62.80 ± 8.84	66.23 ± 8.09
	± 60%	63.02 ± 8.78	66.90 ± 7.86
	± 70%	63.03 ± 8.67	66.82 ± 7.87
	± 80%	61.34 ± 8.62	65.41 ± 7.92
	± 90%	65.68 ± 8.12*	65.82 ± 7.72
Height shift	± 10%	58.82 ± 8.97	67.00 ± 7.98
	± 20%	58.94 ± 8.80	67.12 ± 8.08
	± 30%	61.81 ± 8.74	**67.26 ± 7.87**
	± 40%	**62.03 ± 8.57**	67.23 ± 7.80
	± 50%	61.78 ± 8.42	67.17 ± 7.89
	± 60%	60.21 ± 8.64	66.97 ± 7.94
	± 70%	61.55 ± 8.46	66.69 ± 7.98
	± 80%	60.42 ± 8.19	66.26 ± 7.94
	± 90%	61.52 ± 8.27	67.06 ± 7.58
Rotation	± 3°	57.74 ± 9.37	66.41 ± 8.09
	± 6°	**59.97 ± 9.06**	65.61 ± 8.16
	± 10°	55.40 ± 9.75	65.74 ± 8.15
	± 15°	55.50 ± 9.65	67.03 ± 8.10
	± 45°	54.66 ± 9.62	68.38 ± 8.00
	± 90°	57.62 ± 9.37	**69.86 ± 7.79**
	± 135°	58.60 ± 9.49	68.22 ± 8.07
	± 180°	58.19 ± 9.35	68.78 ± 8.10
Shear	± 3°	59.62 ± 9.05	66.24 ± 8.11
	± 6°	**61.66 ± 8.98**	67.00 ± 8.02
	± 10°	59.42 ± 9.00	67.32 ± 7.90
	± 15°	57.91 ± 9.10	67.11 ± 7.97
	± 45°	59.07 ± 9.80	**68.88 ± 7.74**
	± 90°	56.38 ± 9.3	67.84 ± 7.85
	± 135°	55.22 ± 9.37	67.53 ± 7.91
	± 180°	57.09 ± 8.89	67.67 ± 7.90
Zoom in	0.9, 1	**60.19 ± 8.54**	66.71 ± 8.08
	0.8, 1	59.98 ± 8.53	67.45 ± 8.01
	0.7, 1	57.01 ± 9.46	67.56 ± 8.24
	0.6, 1	55.57 ± 10.07	68.54 ± 8.14
	0.5, 1	57.37 ± 10.30	**68.80 ± 8.25**
	0.4, 1	58.58 ± 10.18	67.26 ± 8.29
	0.3, 1	58.41 ± 10.40	66.54 ± 8.30
	0.2, 1	57.71 ± 10.34	65.54 ± 8.37
	0.1, 1	57.56 ± 10.06	64.05 ± 8.51
Zoom out	1, 1.1	58.70 ± 9.12	65.48 ± 8.17
	1, 1.2	61.64 ± 8.26	66.25 ± 8.09
	1, 1.3	58.99 ± 8.50	65.88 ± 8.03
	1, 1.4	62.21 ± 8.04	66.13 ± 7.98
	1, 1.5	61.83 ± 8.39	66.56 ± 7.86
	1, 1.6	**64.03 ± 8.26**	67.38 ± 7.80
	1, 1.7	60.67 ± 7.90	67.38 ± 7.83
	1, 1.8	62.01 ± 8.20	**67.97 ± 7.63**
	1, 1.9	62.73 ± 8.00	67.91 ± 7.57
	1, 2.0	64.00 ± 8.13	67.97 ± 7.64
Horizontal flip	True	55.89 ± 9.22	67.57 ± 8.11
Vertical flip	True	59.54 ± 8.90	67.23 ± 8.08
Elastic deformation	250, 40	**60.26 ± 8.79**	65.92 ± 8.19
	500, 40	59.17 ± 9.31	65.86 ± 8.18
	1000, 40	57.93 ± 9.12	66.97 ± 8.00
	2000, 40	57.83 ± 8.86	67.88 ± 8.02
	3000, 40	55.89 ± 9.14	**68.17 ± 8.00**
	4000, 40	54.65 ± 9.12	66.96 ± 8.20
	5000, 40	56.55 ± 9.13	65.17 ± 8.36
	6000, 40	55.90 ± 9.37	65.02 ± 8.28
Pixel-based transformations			
Brightness, all channels equally	± 25	59.89 ± 84	66.87 ± 7.66
	± 50	63.27 ± 8.41	66.22 ± 7.74
	± 75	66.79 ± 8.28**	65.17 ± 7.76
	± 100	67.99 ± 8.23**	64.55 ± 7.86
	± 125	68.98 ± 7.90***	63.95 ± 7.87
	± 150	70.07 ± 7.75***	67.25 ± 7.86
	± 175	68.32 ± 7.74**	**67.70 ± 7.88**
Brightness, each channel independently	± 25	71.21 ± 7.69***	67.85 ± 7.84
	± 50	70.90 ± 7.81***	68.28 ± 7.78
	± 75	69.26 ± 8.19***	68.91 ± 7.60
	± 100	69.07 ± 8.26***	69.21 ± 7.51
	± 125	67.86 ± 8.27**	**69.36 ± 7.46**
	± 150	67.86 ± 7.77**	67.07 ± 8.05
	± 175	66.15 ± 8.16*	68.39 ± 7.65
Contrast, all channels equally	0.8, 1.2	58.11 ± 9.35	66.89 ± 7.98
	0.6, 1.4	61.55 ± 8.76	67.31 ± 7.85
	0.4, 1.6	66.17 ± 8.37*	67.92 ± 7.56
	0.2, 1.8	68.38 ± 8.06**	**68.16 ± 7.63**
	0.0, 2.0	60.54 ± 9.43	66.29 ± 8.14
Contrast, each channel independently	0.8, 1.2	71.80 ± 7.61***	**67.68 ± 7.95**
	0.6, 1.4	71.70 ± 7.62***	66.58 ± 7.79
	0.4, 1.6	72.34 ± 7.81***	66.45 ± 7.63
	0.2, 1.8	70.54 ± 7.97***	66.83 ± 7.46
Application-based transformations			
Specular lights	True	59.64 ± 9.06	67.52 ± 7.59
Blurry image	3	**60.32 ± 8.67**	66.14 ± 8.01
	5	58.94 ± 9.37	65.54 ± 8.01
	7	53.61 ± 9.33	64.86 ± 8.05
	9	50.39 ± 9.84**	**66.81 ± 7.93**
	11	51.24 ± 10.02*	64.78 ± 8.12
	13	52.21 ± 9.75*	65.85 ± 8.13
	15	48.41 ± 10.32**	64.91 ± 8.13

Best value for each transformation is indicated in bold

Statistical differences between baseline and the particular case are identified with permutation test

^***^p value < 0.001; **p value < 0.01; *p value < 0.05

**Fig. 3:**
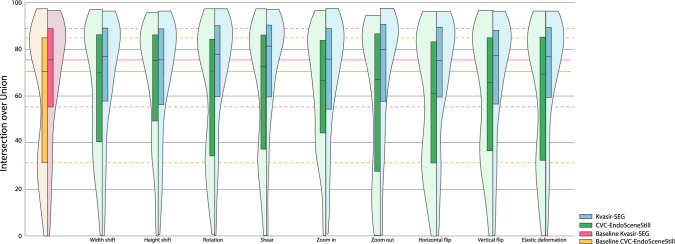
Results for image-based transformations. Ranges with highest mean are shown for each transformation and dataset. Baselines of each dataset are included. Their median and quartiles are prolongated on the background for reference. For the CVC-EndoSceneStill: ± 90% width shift; ± 40% height shift; ± 6° rotation, ± 45° shear; 0.9 zoom in; 0.4 zoom out; (250,40) elastic deformation. For the Kvasir-SEG: ± 20% width shift; ± 30% height shift; ± 90° rotation, ± 45° shear; 0.5 zoom in; 0.2 zoom out; (3000,40) elastic deformation

**Fig. 4:**
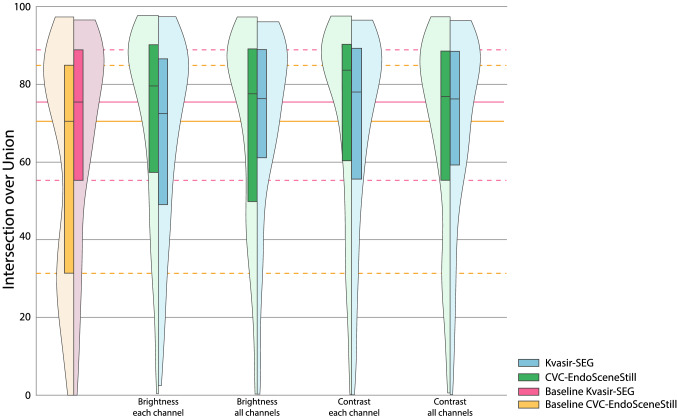
Results for image-based transformations. Ranges with highest mean are shown for each transformation and dataset. Baselines of each dataset are included. Their median and quartiles are prolongated on the background for reference. For the CVC-EndoSceneStill: ± 150 for brightness in all channels equally; ± 25 for brightness in each channel independently; (0.2–1.8) for contrast in all channels equally; and (0.4–1.6) for brightness in each channel independently. For the Kvasir-SEG: ± 175 for brightness in all channels equally; ± 125 for brightness in each channel independently; (0.2–1.8) for contrast in all channels equally; and (0.8–1.2) for brightness in each channel independently

**Fig. 5:**
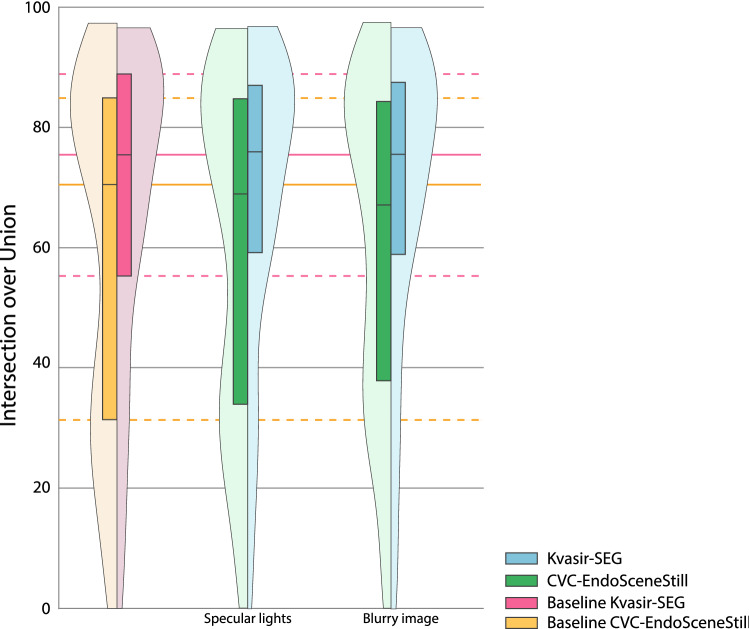
Results for problem-based transformations. Ranges with highest mean are shown for each transformation and dataset. Baselines of each dataset are included. Their median and quartiles are prolongated on the background for reference. For the CVC-EndoSceneStill: 3 for blurry images. For the Kvasir-SEG: 9 for blurry images

Figures 3, 4 and 5 show the range with the highest mean for each transformation for the CVC-EndosceneStill and Kvasir-SEG. Figures for all transformations and ranges can be found in the Supplementary material 1 for CVC-EndoSceneStill dataset and Supplementary material 2 for Kvasir-SEG. All figures show boxplots combined with violin plots, representing the distribution of the results. In these violin plots, the ideal outcome is that the distribution presents a peak at 1. Therefore, the more the distribution looks alike this peak, the better the performance is.

Image-based transformations have different behaviours depending on the dataset, transformation and range. In first place, width and height shift transformations are dependent on the range to either improve or hinder performance of the network in both cases. Only ranges over 40% produce a positive effect, up to 6.59 points, although statistical significance is not achieved in CVC-EndoSceneStill. If Kvasir-SEG is considered, these transformations improve the baseline if small ranges are used, but not significantly. Secondly, rotation and shear results are in all cases under the baseline threshold, reaching 4.43 points decrement in the performance for CVC-EndoSceneStill. On the contrary, these transforms improve performance on Kvasir-SEG in up to 3.41 points, being the greatest improvement in this dataset. Zooming the image has different results depending on whether it is zoom in or out in CVC-EndoSceneStill. Zooming in decreases the performance more than 3.5 points, while zooming out can improve results up to almost 5 points, but significance is not achieved. On the contrary, some ranges from both transforms improve performance in Kvasir-SEG although not significantly. In relation to flipping the image, when CVC-EndoSceneStill is considered, horizontally flipping the image hinders the performance but if flipping is vertical, then performance is increased. In both cases, changes are not significant. On the contrary, both transforms improve performance in Kvasir-SEG, also without statistical significance. Lastly, elastic deformation of the image leads to deterioration of performance of up to 4.45 points in CVC-EndoSceneStill, but improve performance in 1.72 points in Kvasir-SEG.

The second group of transformations modified the pixel-value. On one hand, changes in brightness in CVC-EndoSceneStill, regardless of modifying all channels equally or each channel independently, yield to a better performance of the model of more than 12 points, obtaining significant differences in all cases but two. Similarly, modifying the contrast reached an increment of 13.25 points with respect to the baseline, being this the greatest improvement in all transformations and ranges, and obtaining statistically significant differences for all ranges if channels are modified independently and two out of four if they are equally modified. This behaviour is not so strong in the Kvasir-SEG, while changing brightness and contrast do improve performance in some ranges, significance is not achieved.

Lastly, we analysed transformations based on specific problems of colonoscopy images: adding specular lights and blurring frames. In the first case, including specular lights increased performance in half point and one point regarding the baseline for each dataset, although significance is not achieved in any dataset. On the second case, blurring the image resulted on a significant decrement of up to 10.69 points when compared to the baseline in the case of CVC-EndoScenestill, but only 1.59 points and no significance in Kvasir-SEG.

**Fig. 6:**
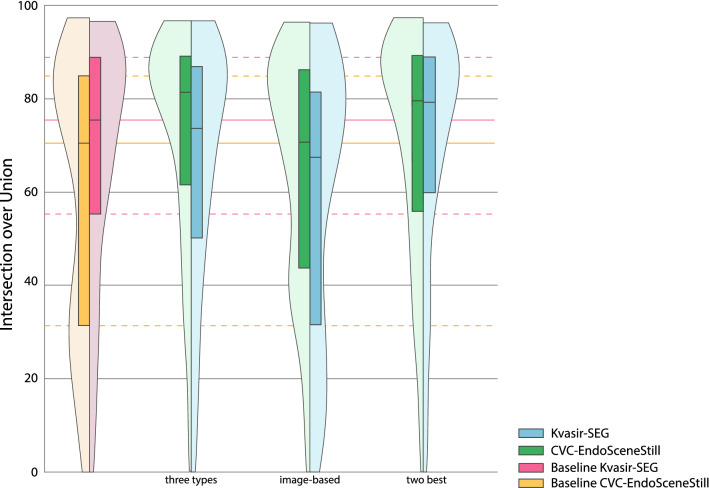
Results for combination of transformations. Baselines of each dataset are included. Their median and quartiles are prolongated on the background for reference. Combination of the transformation and range with highest mean for each one of the three types of transforms for each dataset. For CVC-EndoSceneStill: width at ± 90%, change of contrast: each channel independently, with range [0.4, 1.6], and inclusion of specular lights. For Kvasir-SEG: 90° rotation, change of brightness: each channel independently, with range ± 125, and inclusion of specular lights. Combination of the range with highest mean of the image-based transformations, provided that they improve the baseline result. For CVC-EndoSceneStill: width at ± 90%, height at ± 40%, zoom with range [1, 1.6], and vertical flip. For Kvasir-SEG: width at ± 20%, height at ± 30%, 90° rotation, 45° shear, zoom with range [0.5, 1], vertical flip, horizontal flip, and elastic deformation, with values (3000,40). Combination of the two transformations with higher mean. For CVC-EndoSceneStill: change of contrast: each channel independently, with range [0.4, 1.6] and change of brightness: each channel independently, with range ± 25. For Kvasir-SEG: 90° rotation and 45° shear

Based on these results, we have also analysed combinations of transformation for the different datasets. Results are included in Table 5 and Fig. 6. In all cases for CVC-EndoSceneStill, the mean of these combinations is similar to the transformation with higher mean, but the distributions are improved as the 25 quartile is increased and the standard deviation is minimized. On the other hand, the combination of all image-based transformations hinders the performance, proving that more data augmentation is not always better [24], as only the two image-based transformations with higher mean obtain the best results.

**Table 5:** Mean and standard deviation of combinations analysed

	CVC-EndoSceneStill		Kvasir-SEG	
	Transformations	IoU on test set	Transformations	IoU on test set
Baseline	None	59.10 ± 9.35	None	66.45 ± 8.08
Transformation and range with highest mean for each one of the three types of transforms	Width at ± 90%	72.30 ± 7.26***	90° rotation	65.53 ± 7.98
	Change of contrast: each channel independently, with range [0.4, 1.6]		Change of brightness: each channel independently, with range ± 125	
	Inclusion of specular lights		Inclusion of specular lights	
Range with highest mean of the image-based transformations, provided that they improve the baseline result	Width at ± 90%	65.19 ± 7.81*	Width at ± 20%	57.97 ± 9.21**
	Height at ± 40%		Height at ± 30%	
	Zoom with range [1, 1.6]		90° rotation	
	Vertical flip		45° shear	
			Zoom with range [0.5, 1]	
			Vertical flip	
			Horizontal flip	
			Elastic deformation, with values (3000,40)	
The two transformations with higher mean	Change of contrast: each channel independently, with range [0.4, 1.6]	70.50 ± 7.69***	90° rotation	69.24 ± 7.85
	Change of brightness: each channel independently, with range ± 25		45° shear	

Statistical differences between baseline and combination are identified with permutation test

^***^p value < 0.001; **p value < 0.01; *p value < 0.05

## Discussion and conclusion

Data augmentation is a useful tool to increase the number of training samples when the available dataset is scarce, a situation that is well-known when using medical images. The effect of different transformations usually applied in data augmentation for polyp segmentation has yet to be rigorously analysed. In this work, we have found that although image-based transformations are usually applied in the state of the art, pixel-based transformations produce better results for CVC-EndoSceneStill. These transformations modify the particular value of the pixel, so the model is invariant to colour information, which improves its generalization capacity. On the other hand, Kvasir-SEG benefits to a greater extent from the image-based transformations.

In the light of the results, four new groups of transformations can be established:

1. Transformations that always improve the performance in CVC-EndoScenStill and Kvasir-SEG: vertical flip, changes on brightness for each channel independently, changes on contrast (all channels equally and each channel independently) and inclusion of specular lights. All these transformations improve the performance over the baseline, although statistical significance is mainly found in changes of brightness and contrast in CVC-EndoSceneStill.
2. Transformations that always hinder the performance in CVC-EndoScenStill and Kvasir-SEG: elastic deformation and blurry frames (mean filter). While blurry frames could be expected to minimize the performance as they reduce the details in the image, elastic deformation might have been expected to improve performance. Although blurry frames are a common situation during a live colonoscopy, the inclusion of mean filter as transformation for data augmentation does not improve the final performance of the model. This is probably explained by the use of databases, where frames are previously selected and not blurry frames are included.
3. Transformations whose effect on performance depends on the selected range in CVC-EndoScenStill and Kvasir-SEG: height and width shifts, as well as zoom in and out. In the first two cases, ranges over 40% do contribute to improve performance, while under the threshold either the transformation does not add improvement or decrement the performance. On the other hand, zoom behaviour also depends on the range. Smaller ranges of zoom in and larger ranges of zoom out improve the performance over the baseline, although not always significantly. One reason for the performance of the zoom in might be grounded on the low quality of the original images, resulting in blurry zoomed images. Therefore, when using them for data augmentation, it is recommended to carefully check whether the range is suitable or not.
4. Transformations whose effect on performance depends on the dataset, CVC-EndoScenStill or Kvasir-SEG: This relates mainly to rotation, shear and changes on brightness for all channels equally, and, to a lesser extent, horizontal flip. This might be due to differences in polyp size, void area, brightness and contrast in the images of the two datasets.

In summary, CVC-EndoSceneStill is more prone to benefits of data augmentation if pixel-based transformations are used, as the histogram is flatter, and images are darker than in Kvasir-SEG. On the contrary, image-based transformations appear to be more suitable in Kvasir-SEG, where the void area is smaller, and the polyp occupy a greater area of the valid image. Lastly, problem-based transformations behave similarly in both datasets, as they are rooted on the endoscopic image acquisition. It is also important to mention that the baseline of Kvasir-Seg showed already a better performance than CVC-EndosSceneStill, giving less room for improvement to data augmentation.

There are different approaches to overcome the scarce labelled datasets in medical imaging. On the one side, and in order to increase the size of the training set, a first approach would be to increase the number of annotated samples by experts. In this regard, efforts are been focused on developing tools which facilitates the manual annotation of images, such as GTCreatorTool [22], which is a flexible annotation tool which minimizes annotation time and allows for sharing annotations among experts. Beyond the transformations analysed in this paper, other alternatives would be to add polyps in nonpolypoid samples [41] or more advances approaches such as emulating data augmentation during learning by the image generation through a hetero-encoder [42]. On the other hand, it would be possible to explore alternatives to supervised training, which already seems to provide good results with self-supervised learning [43] or similarity-based active Learning [44].

There are limitations in this study that must be acknowledged. Ideally, it would be necessary to independently analyse all combinations. Since that would mean almost 6 million experiments, alternatives such as AutoAugment [7] or Smart Augmentation [10] would be more suitable for identification of the best combination of transformations. Another possibility could be the application of Bayesian methods [45] or coordinate ascent optimization [46, 47] taking the optimal setting of each transform to identify the best combination. Future work should place emphasis on applying any of these alternatives to the particular field of polyp segmentation. Another limitation is the fact that the experiments have not pursued the best model, so training has been stopped at 15 epochs. It might be possible that with a more extensive training some of the transformations could have showed better results. Nevertheless, 15 epochs is enough training to establish the tendency of the model performance when finetuning it with a small database.

Further research is also possible in this line of work. Future works might focus on the effect of data augmentation on other segmentation approaches, such as the fuzzy C-mean clustering, which has shown good preliminary results on the Kvasir-SEG database [20].

In conclusion, this study shows that different transformations and ranges lead to differences in model performance. Despite not being so frequent as the other types, pixel-based transformations show a great potential to improve polyp segmentation. Augmenting colour variability when training the model allows for a better generalization of the model resulting in better prediction. On the other hand, image-based transformations and their ranges should be carefully selected to not hinder the model performance and obtain the expected benefits of data augmentation.

## Electronic supplementary material

Below is the link to the electronic supplementary material.

Supplementary file1 (PDF 1257 kb)

Supplementary file2 (PDF 1248 kb)
