# Supplementary material for: Unravelling the effect of data augmentation transformations in polyp segmentation
Source: Int J Comput Assist Radiol Surg. 2020 Sep 28;15(12):1975–88. doi: 10.1007/s11548-020-02262-4 (PMC7671995; doi:10.1007/s11548-020-02262-4)
Supplement: Supplementary file 1 — Supplementary file1 (PDF 1257 kb) [file 11548_2020_2262_MOESM1_ESM.pdf]

Unravelling the effect of data augmentation transformations in polyp segmentation

International Journal of Computer Assisted Radiology and Surgery

Luisa F. Sánchez-Peralta*, Artzai Picón, Francisco M. Sánchez-Margallo, J. Blas Pagador
* Jesús Usón Minimally Invasive Surgery Centre. Cáceres (Spain)
<lfsanchez@ccmijesususon.com>

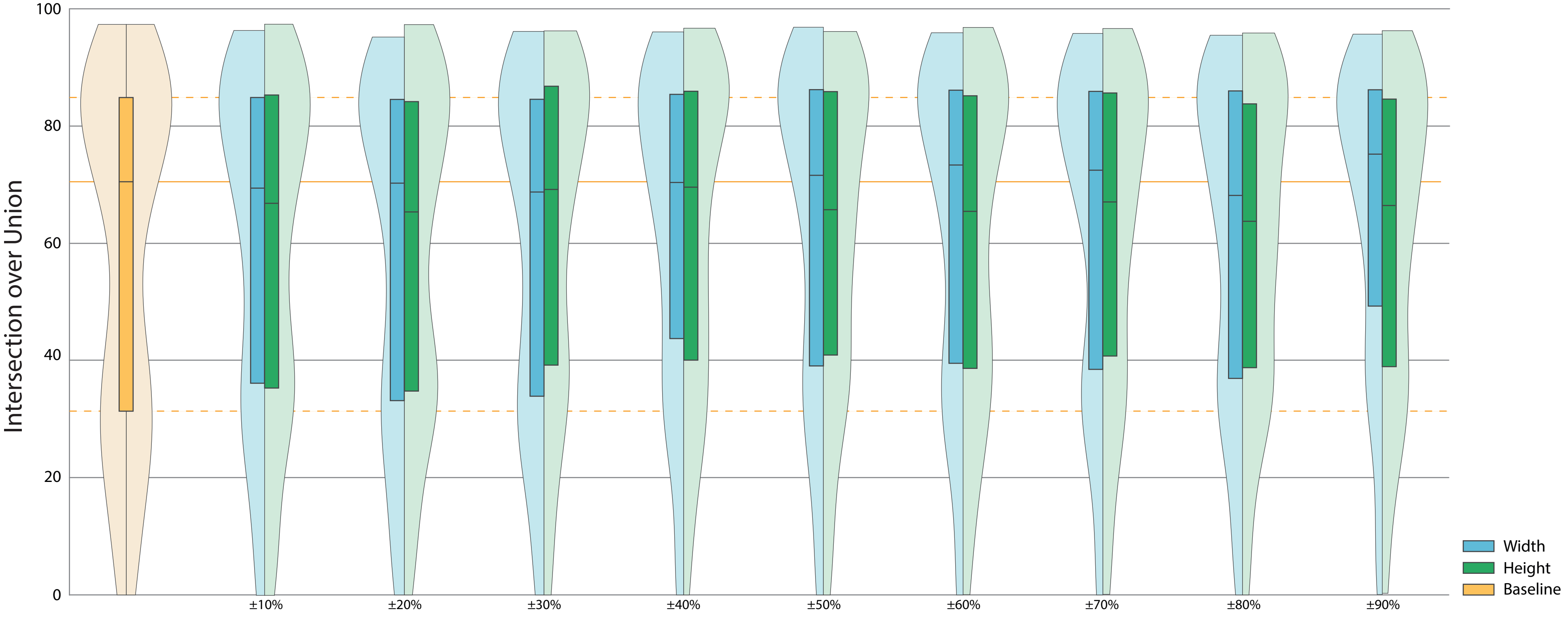

Fig S1.1. Results for width and heigh shifts for CVC-EndoSceneStill. Baseline is included.
Its median and quartiles are prolongated on the background for reference.

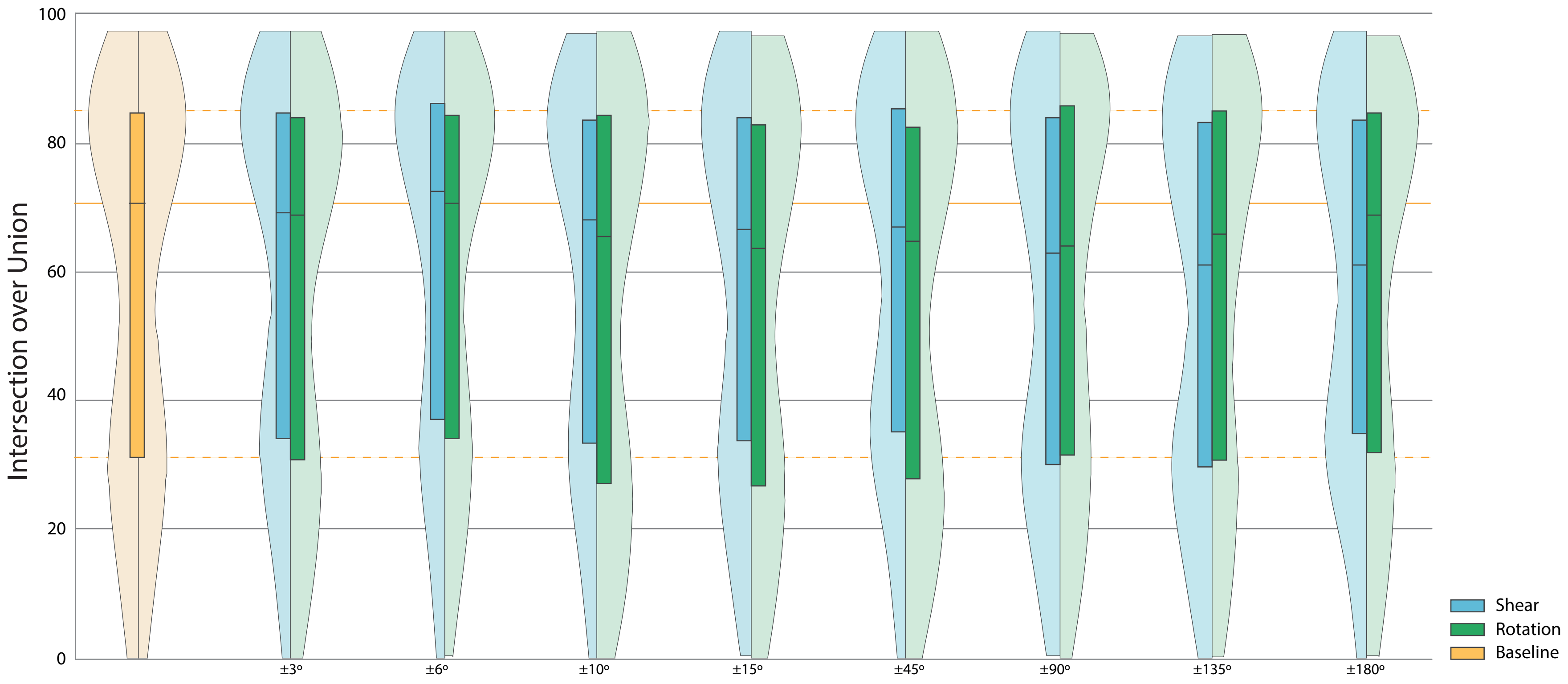

Fig S1.2. Results for rotation and shear for CVC-EndoSceneStill. Baseline is included. Its median and quartiles are prolongated on the background for reference.

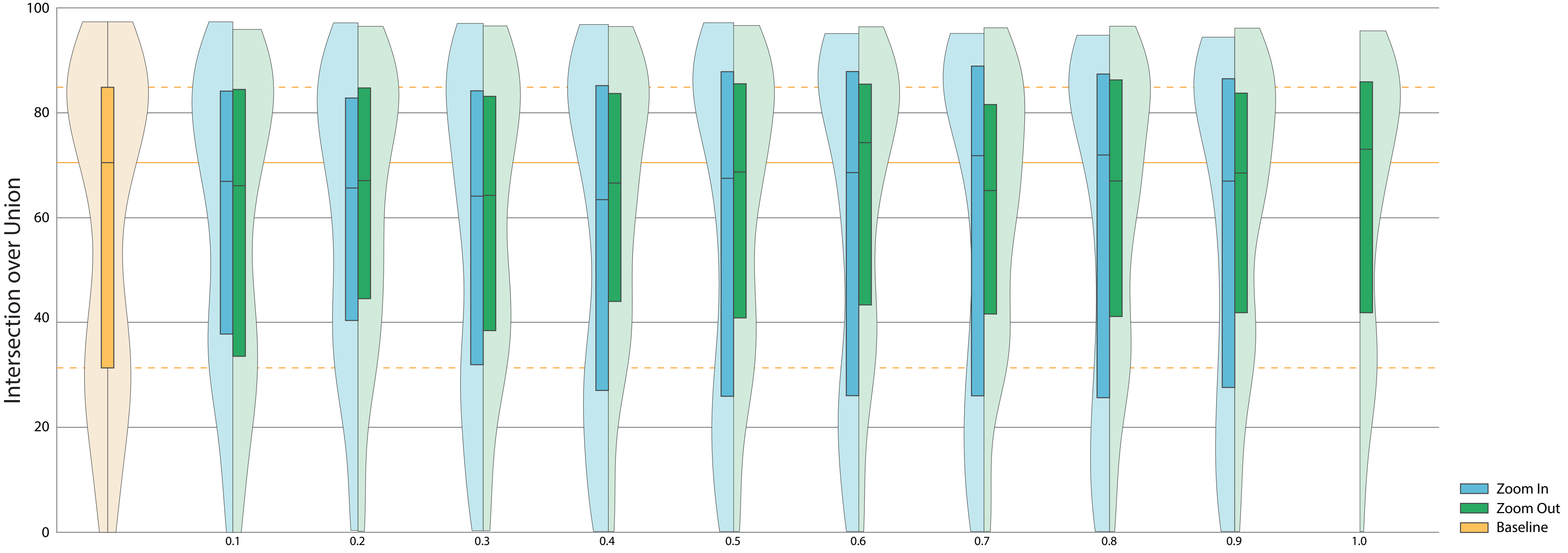

Fig S1.3. Results for zoom in and out for CVC-EndoSceneStill. Baseline is included. Its median and quartiles are prolongated on the background for reference.

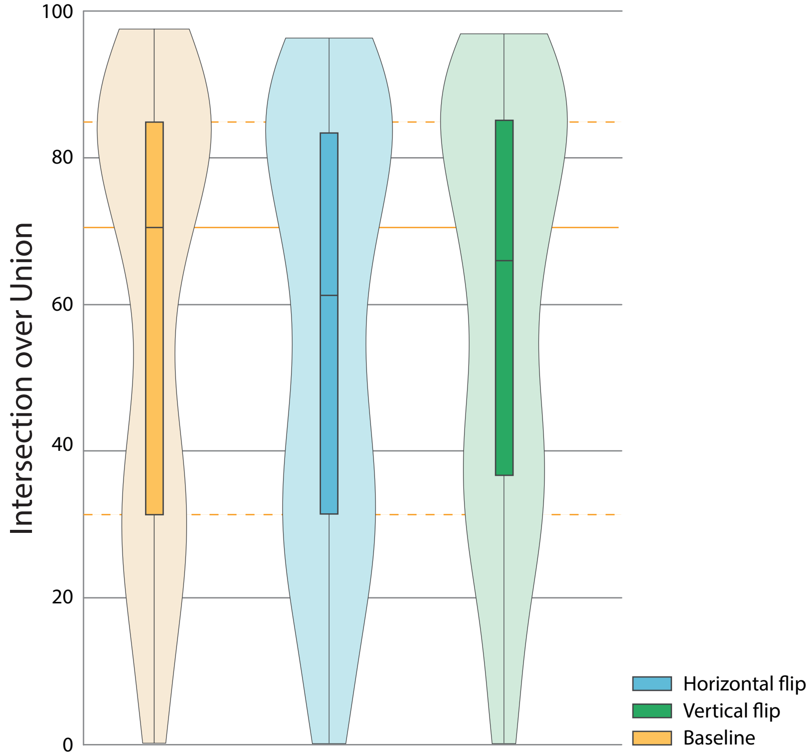

Fig S1.4. Results for horizontal and vertical flips for CVC-EndoSceneStill. Baseline is included. Its median and quartiles are prolongated on the background for reference.

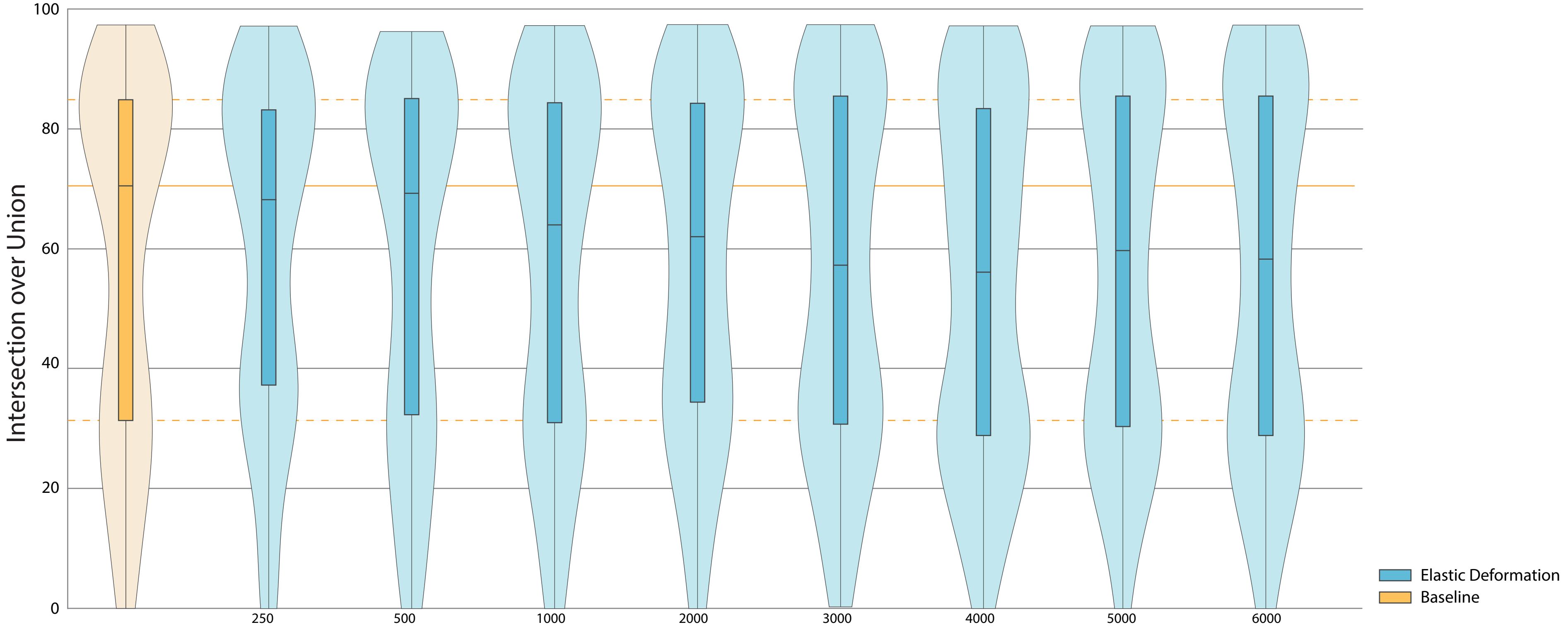

Fig S1.5. Results for elastic deformation for CVC-EndoSceneStill. Baseline is included. Its median and quartiles are prolongated on the background for reference.

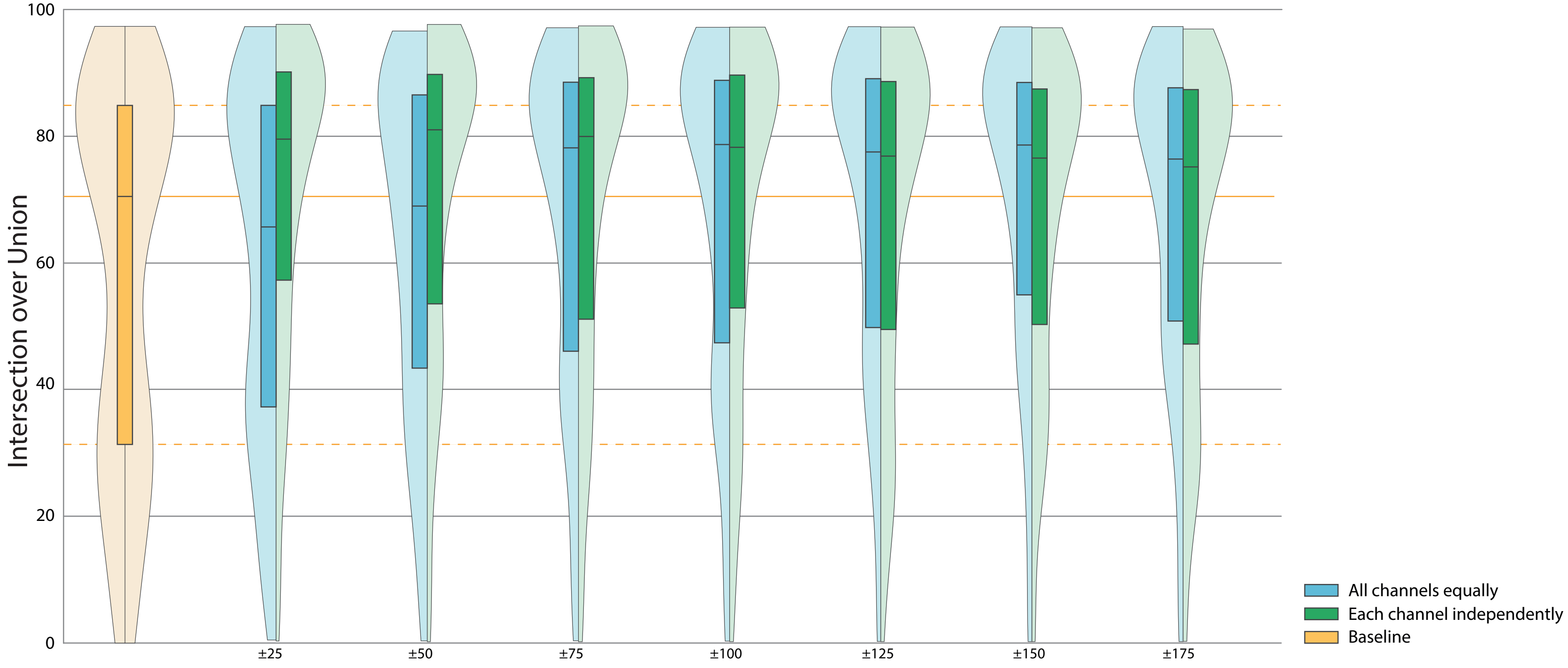

Fig S1.6. Results for changes in brightness for CVC-EndoSceneStill. Baseline is included. Its median and quartiles are prolongated on the background for reference.

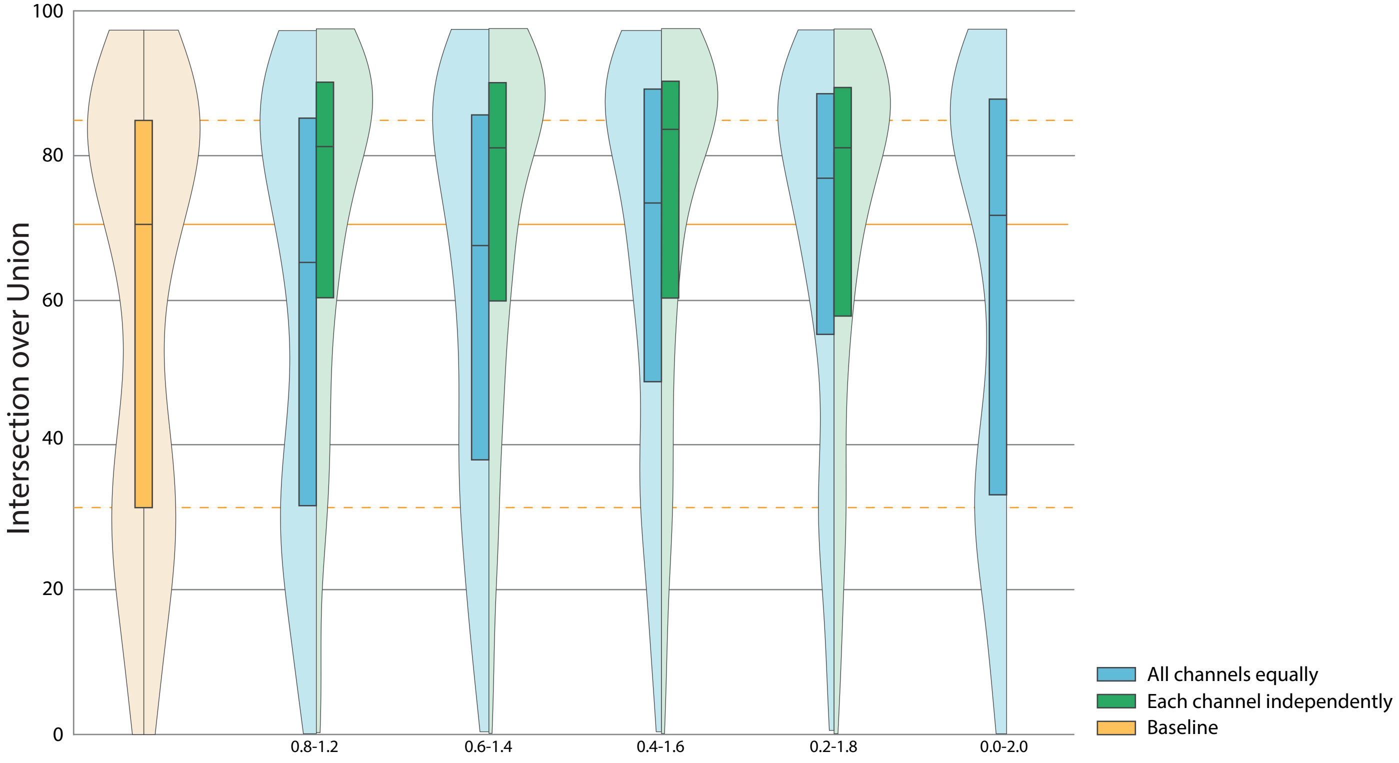

Fig S1.7. Results for changes in contrast for CVC-EndoSceneStill. Baseline is included. Its median and quartiles are prolongated on the background for reference.

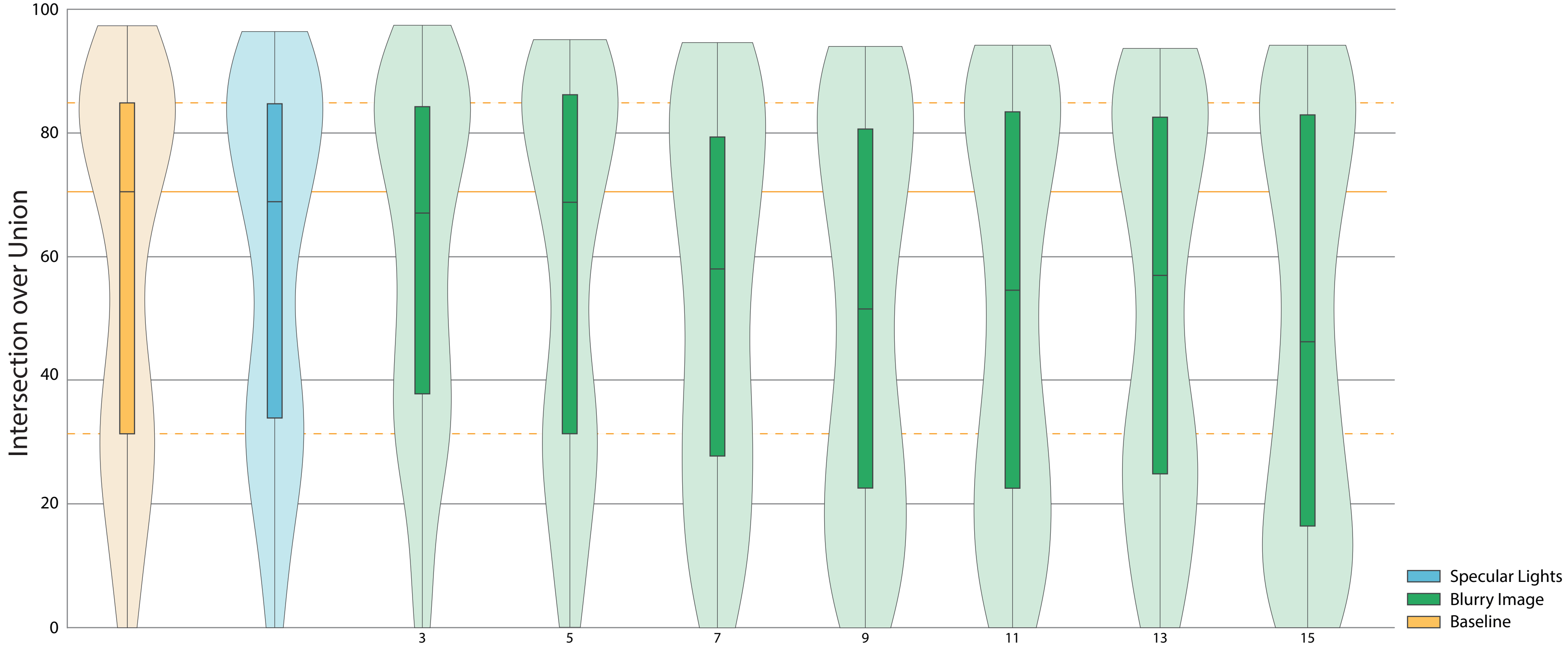

Fig S1.8. Results for inclusion of specular lights and blurry frames (mean filter) for CVC-EndoSceneStill. Baseline is included. Its median and quartiles are prolongated on the background for reference.
